# Remarks upon a Case of Hydrocele, Which Terminated Fatally

**Published:** 1807-12

**Authors:** J. L. Green

**Affiliations:** Lewisham


### *Remarks upon a Case of Hydrocele, which terminated fatally.*

EVERY medical man must have observed, that extraordinary Cases do now and then occur, which in their outset, appear not to require any great skill or judgment to conduct for the safety of the patient; yet, in their progress, from some unknown or accidental circumstances, a sudden change takes place, baffling every endeavour and every art, and that their termination is sometimes as fatal, as their progress has been rapid.

So, on the contrary, cases, which in their very commencement appear to require both judgment and experience to manage for the safety of the patient, shall do well, with very little, and sometimes without the smallest assistance from art; nay, that they frequently end favourably, in spite of every obstacle, under the very worst kind of treatment from ignorance and quackery. In one case case the attending party meets with undeserved censure; in the other with unmerited praise: The multitude judge from the event, and if an operation turns out unsuccessful, the operator will be commonly blamed; for they are incapable of discriminating between the post hoc and the propter hoc; and any attempt at elucidation is looked upon by them, as only done to vindicate what they judge erroneous practice; but suffer me to ask, who would practice surgery, if its professors were amenable to obloquy, whenever their endeavours were not crowned with success?

I beg leave to exemplify the first of these Remarks, by the relation of an uncommou case; of successful cases there are plenty on record, but there is naturally a reluctance about the human mind to relate unsuccessful cases, from fear of incurring the censures of ill-nature, or of ignorance.

An elderly man (about 67 years of age) consulted me concerning a large swelling which he had in his scrotum, on the left side; upon attentively examining it, I declared it to be a collection of water; he then said he had been informed so before, and had been advised to have it let out. From his conversation I learnt, that he had shewn it to several people, empirics as well as regular practitioners, who mostly agreed, that it was, to use his own expression, a watery rupture. It had been forming for three years, and had been gradually increasing, and was now become extremely troublesome from its weight and magnitude, occasioning a constant aching pain in the loins. I recommended the drawing off the water, and he immediately consenting, I punctured the anterior part of the tunica vaginalis, and drew off half a common chamber-pot full of pale yellow serum. From a state of pain and uneasiness he became instantly easy, and expressed great satisfaction. I dressed the puncture superficially with a morsel of lint, and a bit of adhesive plaster, and suspended the parts in a bag truss: and having done this, I expected no further trouble about it.

The puncture was made in the afternoon (29th of October) and the patient remained perfectly easy for several hours; however, pain came on in the night, and early in the morning he sent for me. I found him up, and complaining of excessive pain in the loins, and at the bottom of the scrotum, which appeared a little inflamed. I ordered him to bed, gave him an opening draught, had the parts fomented with an antiseptic fomentation, a soft poultice poultice of linseed meal applied; he soon got relief in his back from lying in an horizontal posture; but the pain continued at the bottom of the scrotum; by the evening of this day, the inflammation had rapidly extended itself over the whole scrotum, and there appeared a spot, about the size of a six-pence, sphacelated on the right side, and, it is worthy of remark, that the mortification began at a considerable distance from where the puncture had been made. The man became extremely agitated and alarmed, and, at my desire, Doctor Leith of Greenwich was called in, who after emptying the bowels, prescribed bark, opium, &c. and allowed him wine; the fomentation and poultices were assiduously continued. We went on this way until the fourth day; but, notwithstanding all our endeavours, the sphacelation continued increasing, and by this time had extended over the whole of the integuments and cellular membrane of the scrotum, which was much distended, but not very painful to the touch, and the cuticle had peeled off in more than one place; he had had one, and sometimes two motions every day; he had a very weak quick pulse, and a dry furred tongue; wine, bark, and opiates were liberally administered. On the fifth day, the mortified parts began to slough, and the tumefaction over the scrotum began to subside; but now the mischief had extended itself to the pubes and to the penis, which was much swollen, and the prepuce began to sphacelate. As this was altogether an uncommon case, and the family wishing all the assistance they could get, the attendance of Mr. Ashley Cooper was requested, who obligingly came to us at night. He recommended the parts to be fomented with hot vinegar; the poultice to be made with yeast; to continue the bark, &c. and also to give him 10 grains of pulv. ipecacoan. comp. which was repeated in the morning. From the quantity of wine, brandy and opium, that the patient had taken, he passed a night with tolerable ease, and towards morning slept a good deal; but as the effects of these went off, he sunk rapidly, and died between ten and eleven at night, on the sixth day.

From the rapid progress, and fatal termination of this case, I confess, that I was not without apprehension, that there was something more about it than simple hydrocele, and, perhaps, that it might be complicated with hernia, or diseased testis, and therefore I was very solicitous to examine the parts; and having obtained permission, I did so. From the dissection, however, I learnt little. There was about two spoonsfull of whitish coloured coagulated serum in the sac; there was no hernia; but the bag extended quite up to the abdominal muscles; the testicle lay at the bottom where it was attached, and appeared undiseased, except partaking of the inflammatory appearance of the surrounding parts; the tunica vaginalis itself was much thickened, and corrugated, and the vessels on its internal surface were much distended with blood, and appeared as if they had been injected; the cellular membrane of the scrotum was full of serum and air.

This man unquestionably must have been of a bad habit, but he appeared hearty for a man of his years, and went constantly about.

It is one of those unhappy and lamentable cases which no human foresight could prevent; nor I believe any surgical skill alleviate or avert; the mere narrating, however, of an unfortunate case is of little use, unless we can draw a lesson from it to regulate our future conduct and practice; and this one ought surely to serve as a caution to the younger part of the profession, never to undertake any operation, however trifling it may appear, without the approbation of some professional man of established reputation to sanction their proceedings; for surely nothing can be more easy than puncturing a distended tunica vaginalis, and yet in the case above related, how unexpected, and how fatal was the termination.

I am, &c.

J. L. GREEN.

*Lewisham, 7 Nov.* 1807.

